# Cardiomyocyte-Specific bcl11b Knockout Causes Left Ventricular Noncompaction by Dysregulating pou3f2 and Titin

**DOI:** 10.3390/ijms27167199

**Published:** 2026-08-12

**Authors:** Wei Bi, Xiaoxi Luo, Yaqi Lv, Lifeng Liu, Youshi Chen, Chenxi Li, Jiani Fu, Shijia Hu, Jianfeng Wang, Xing Chang, Hongjun Shi

**Affiliations:** 1 School of Life Science, Fudan University, Shanghai 200433, China; biwei@westlake.edu.cn; 2 Key Laboratory of Growth Regulation and Translational Research of Zhejiang Province, School of Life Sciences, Westlake University, Hangzhou 310030, China; 3 Westlake Laboratory of Life Sciences and Biomedicine, Hangzhou 310030, China; 4 Institute of Basic Medical Sciences, Westlake Institute for Advanced Study, Hangzhou 310030, China; 5 Centro Nacional de Investigaciones Cardiovasculares, 28029 Madrid, Spain

**Keywords:** Bcl11b, LVNC, Pou3f2, Titin

## Abstract

Left ventricular noncompaction (LVNC) is a cardiomyopathy characterized by excessive trabeculation and deep intertrabecular recesses, yet its molecular mechanisms remain poorly understood. Here, we identify Bcl11b as a novel regulator of cardiomyocyte (CM) growth and ventricular wall maturation. CM-specific deletion of *Bcl11b* in mice recapitulates key LVNC features, including increased noncompacted-to-compacted ratio, impaired compact layer expansion, reduced CM proliferation and size, and systolic dysfunction. Mechanistically, Bcl11b deficiency leads to marked upregulation of *Pou3f2*, a transcriptional repressor that further suppresses *Titin* (*TTN*) expression. Loss of *Bcl11b* disrupts sarcomere integrity and reduces TTN protein levels, while forced *Pou3f2* overexpression similarly represses TTN. Notably, heterozygous loss of *Pou3f2* rescues the LVNC phenotype in *Bcl11b*-deficient hearts, restoring CM growth and *TTN* expression. Our findings identify a critical relationship among Bcl11b, Pou3f2 and TTN that controls CM proliferation and hypertrophic maturation during cardiac development. Dysregulation of this regulatory network impairs ventricular compaction and contributes to the pathogenesis of LVNC, providing new mechanistic insights into disease pathogenesis and highlighting potential therapeutic targets.

## 1. Introduction

Left ventricular noncompaction (LVNC) is a cardiomyopathy defined by excessive trabeculation and deep intertrabecular recesses within the ventricular wall [1,2]. It ranks as the third most common cardiomyopathy, with an estimated prevalence of 0.01–0.3% in adults and up to 9.2% among children diagnosed with cardiomyopathies [3,4]. The clinical presentation varies widely, from asymptomatic individuals to those suffering from progressive heart failure, life-threatening arrhythmias, thromboembolic events, and sudden cardiac death. Despite growing recognition of LVNC as a distinct clinical entity, the molecular pathways driving its pathogenesis remain poorly understood.

Ventricular trabeculation and compaction are fundamental morphogenetic processes that shape the ventricular walls during heart development. Trabeculation begins when the ventricular chambers balloon outward from the primitive heart tube, with cardiomyocytes (CMs) forming a complex meshwork of finger-like projections into the lumen [5,6]. This sponge-like network provides essential mechanical strength to the developing ventricular wall before the establishment of coronary circulation. Subsequently, the compact layer undergoes substantial proliferative expansion, gradually thickening and integrating with the adjacent trabecular meshwork to form a mature, robust ventricular wall [7]. Failure of proper compact layer growth, or conversely excessive trabecular proliferation, can lead to LVNC [8]. Reciprocal signals between endocardium and myocardium orchestrate these events. Notably, NOTCH and NRG signaling have been identified to be the master regulators of trabecular formation by controlling ECM degradation and synthesis that are critical for trabecular rearrangement and growth [6]. Mouse models with disrupted Notch/Nrg signaling, including *Mib1*, *Fkbp1a*, and *Nkx2-5* mutants, recapitulate LVNC features such as hypertrabeculation, reduced compaction, and systolic dysfunction [9,10,11,12].

Despite these insights, Notch/Nrg pathway mutations are rarely found in LVNC patients, suggesting other mechanisms predominate [13]. More than 40 genes have been linked to LVNC, with mutations in sarcomere genes, particularly *MYH7*, *MYBPC3*, *ACTC1*, and *Titin* (*TTN*), accounting for over 40% of identified variants; these mutations are also frequently associated with dilated cardiomyopathy (DCM) and hypertrophic cardiomyopathy (HCM) phenotypes [14,15]. Truncating *TTN* variants (*TTNtv*) are among the most frequent genetic causes, underscoring titin’s essential role in ventricular integrity [16]. Homozygous deletion of *TTN* in mice caused embryonic lethality while heterozygous *TTNtv* mice showed normal cardiac function [17]. Patient-mimicking mutants generally fail to display classic LVNC. Instead, they develop dilated cardiomyopathy-like features dominated by progressive systolic dysfunction without striking trabecular abnormalities [17]. This phenotypic divergence has greatly hindered mechanistic understanding of *TTN*-mediated LVNC.

In this study, we identify Bcl11b as a novel regulator of CM gene expression and CM growth. Loss of *Bcl11b* function upregulates *Pou3f2* and reduces *TTN* expression, thereby recapitulating key LVNC features, including excessive trabeculation, deep recesses, and impaired contractility. These findings provide new insights into the molecular mechanisms by which Bcl11b and its downstream targets, Pou3f2 and TTN, regulate cardiac development and contribute to congenital heart disease (CHD).

## 2. Results

### 2.1. CM-Specific Knockout Bcl11b Causes LVNC During Heart Development

**Figure 1:**
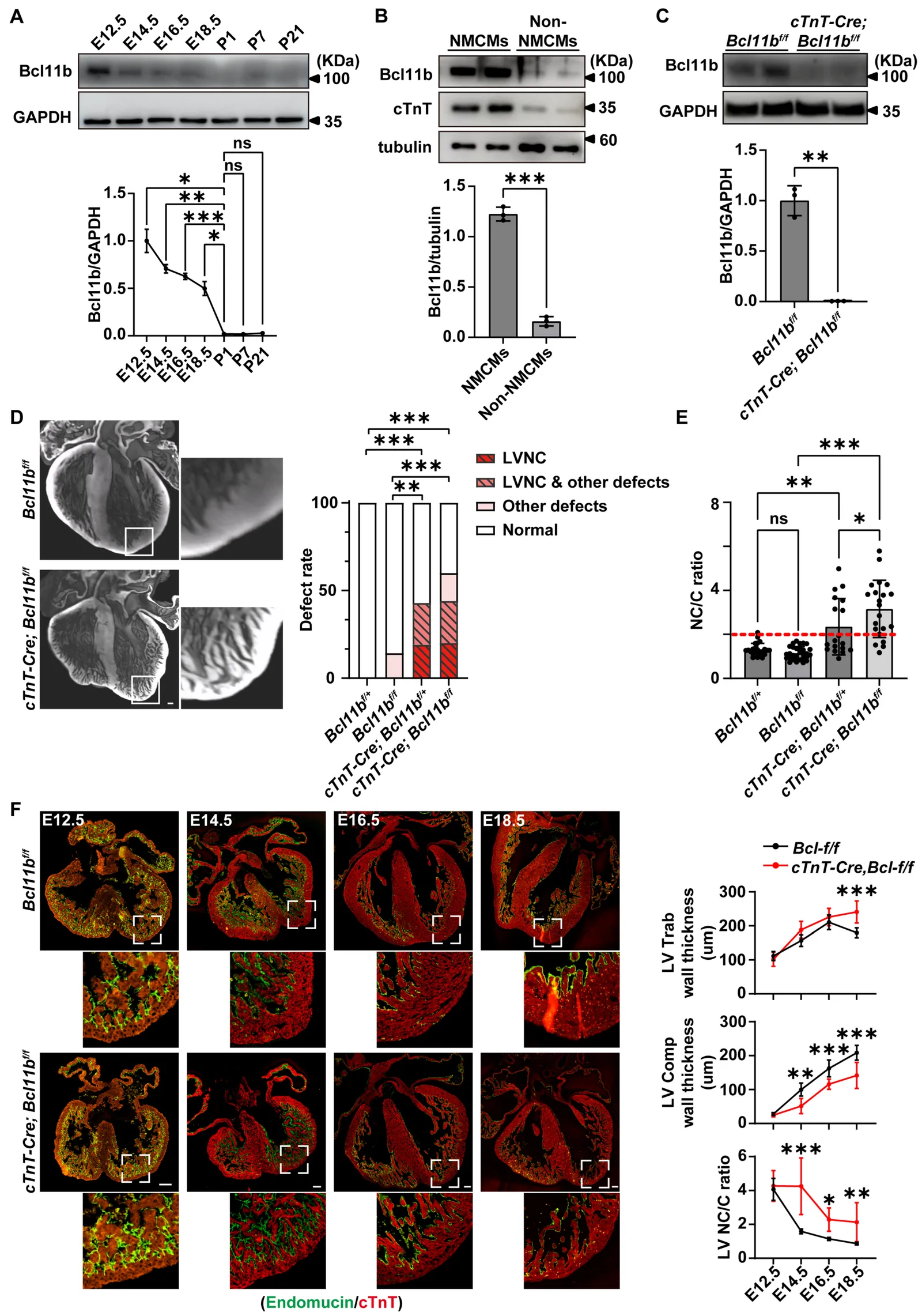
Bcl11b expression is enriched in cardiomyocytes and cardiomyocyte-specific knockout of *Bcl11b* leads to LVNC. (**A**) Bcl11b expression levels in the mouse heart ventricles at different developmental stages. *n* = 3–4 hearts/group. (**B**) Bcl11b expression in neonatal mouse cardiomyocytes (NMCMs) and non-cardiomyocytes (non-NMCMs). *n* = 3 hearts/group. (**C**) Bcl11b protein expression in the left ventricle tissue of control (*Bcl11b^f*/*f^*) and cardiomyocyte-specific knockout (*cTnT-Cre*; *Bcl11b^f*/*f^*) mice. *n* = 3 hearts/group. (**D**) Lightsheet images and quantification data of defect incidence in E18.5 embryonic hearts from offspring of *cTnT-Cre*; *Bcl11b^f/+^* × *Bcl11b^f*/*f^* crosses. The inset (**right**) shows a magnified view of the dashed region. *n* = 21–28 hearts/group. (**E**) Quantification data of the trabecular-to-compact myocardial thickness ratio (NC/C) in the left ventricle (LV) of E18.5 mice with different genotypes, the red dashed line indicates the cutoff value of NC/C ratio = 2. *n* = 18–29 hearts/group. (**F**) Changes in LV trabecular thickness, compact myocardial thickness, and NC/C in heart sections from *Bcl11b^f*/*f^* and *cTnT-Cre*; *Bcl11b^f*/*f^* mice at E12.5, E14.5, E16.5, and E18.5. *n* = 3–13 hearts/group. Scale bar = 100 μm. Means ± SD are plotted; statistics were performed using Welch’s unpaired *t* test (**B**,**C**), the chi-square test with Bonferroni correction for multiple comparisons (**D**), Welch’s ANOVA (**A**) one-way (**E**) or two-way ANOVA followed by multiple comparisons tests (**F**). * *p* < 0.05, ** *p* < 0.01, *** *p* < 0.001; ns, non-significant.

During mouse heart development, *Bcl11b* expression peaked at embryonic day 12.5 (E12.5) and then declined sharply in left ventricle after birth (Figure 1A), suggesting an important role during the embryonic stage. In the left ventricle, Bcl11b was predominantly expressed in CMs (Figure 1B,C) and localized to CM nuclei (Figure S1A). To investigate the function of *Bcl11b* in heart development, we generated specific conditional knockout mice by crossing *Bcl11b^f*/*f^* mice with *Nkx2.5-Cre*, *Nfatc1-Cre* and *cTnT-Cre* lines to ablate *Bcl11b* in cardiac progenitor cells [18], endocardial-derived cells [19] and CMs [20], respectively. At E18.5, *Nfatc1-Cre*; *Bcl11b^f*/*f^* mice exhibited no obvious cardiac abnormalities. In contrast, *Nkx2.5-Cre*; *Bcl11b^f*/*f^* mice and *cTnT-Cre*; *Bcl11b^f*/*f^* mice displayed a comparable 50% incidence of cardiac defects, with the LVNC being the predominant phenotype (Figure 1D and Figure S1B,C). Both *cTnT-Cre*; *Bcl11b^f*/*f^* (CM-KO) and *cTnT-Cre*; *Bcl11b^f/+^* (CM-KD) mice exhibited increasing noncompacted to compacted myocardial thickness ratio (NC/C) compared to wild type (WT) littermates, with a more pronounced increase observed in CM-KO mice (Figure 1E). Histological analysis further revealed that impaired expansion of the compact myocardium from E14.5 onward was the primary contributor to the increased NC/C ratio and ventricular hypertrabeculation observed in *cTnT-Cre*; *Bcl11b^f*/*f^* mice at E18.5 (Figure 1F).

A proportion of the *Nkx2.5-Cre*; *Bcl11b^f*/*f^* mice and *cTnT-Cre*; *Bcl11b^f*/*f^* mice died within the first 3 days after birth. Genotype analysis at postnatal day 3 (P3) revealed that the survival frequency of *Nkx2.5-Cre*; *Bcl11b^f*/*f^* mice significantly deviated from the expected Mendelian ratio, whereas no significant deviation was observed in *cTnT-Cre*; *Bcl11b^f*/*f^* mice (Figure S1D). These findings suggest that, in addition to its essential role in CMs, *Bcl11b* is also required in non-CM derivatives of the *Nkx2.5* lineage, and its deletion in these cells results in partial perinatal lethality.

The surviving *cTnT-Cre*; *Bcl11b^f*/*f^* mice exhibited significantly reduced heart weight (HW), tibia length (TL), and the HW/TL ratio compared with control littermates at P21 (Figure S2A–D). Wheat germ agglutinin (WGA) staining further revealed a marked reduction in CM size (Figure S2A,E), indicating impaired CM growth following *Bcl11b* deletion. Further echocardiographic analysis at both P21 and 8–10 weeks of age demonstrated impaired systolic function accompanied by ventricular wall thinning (Figures S2F–P and S3A–K). Collectively, these findings suggest that Bcl11b is an essential transcription factor for CM growth and cardiac maturation and its deletion in CMs (*cTnT-Cre*; *Bcl11b^f*/*f^*) results in LVNC accompanied by progressive systolic dysfunction.

### 2.2. Absence of Bcl11b Disrupts CM Proliferation and Physiological Hypertrophy

**Figure 2:**
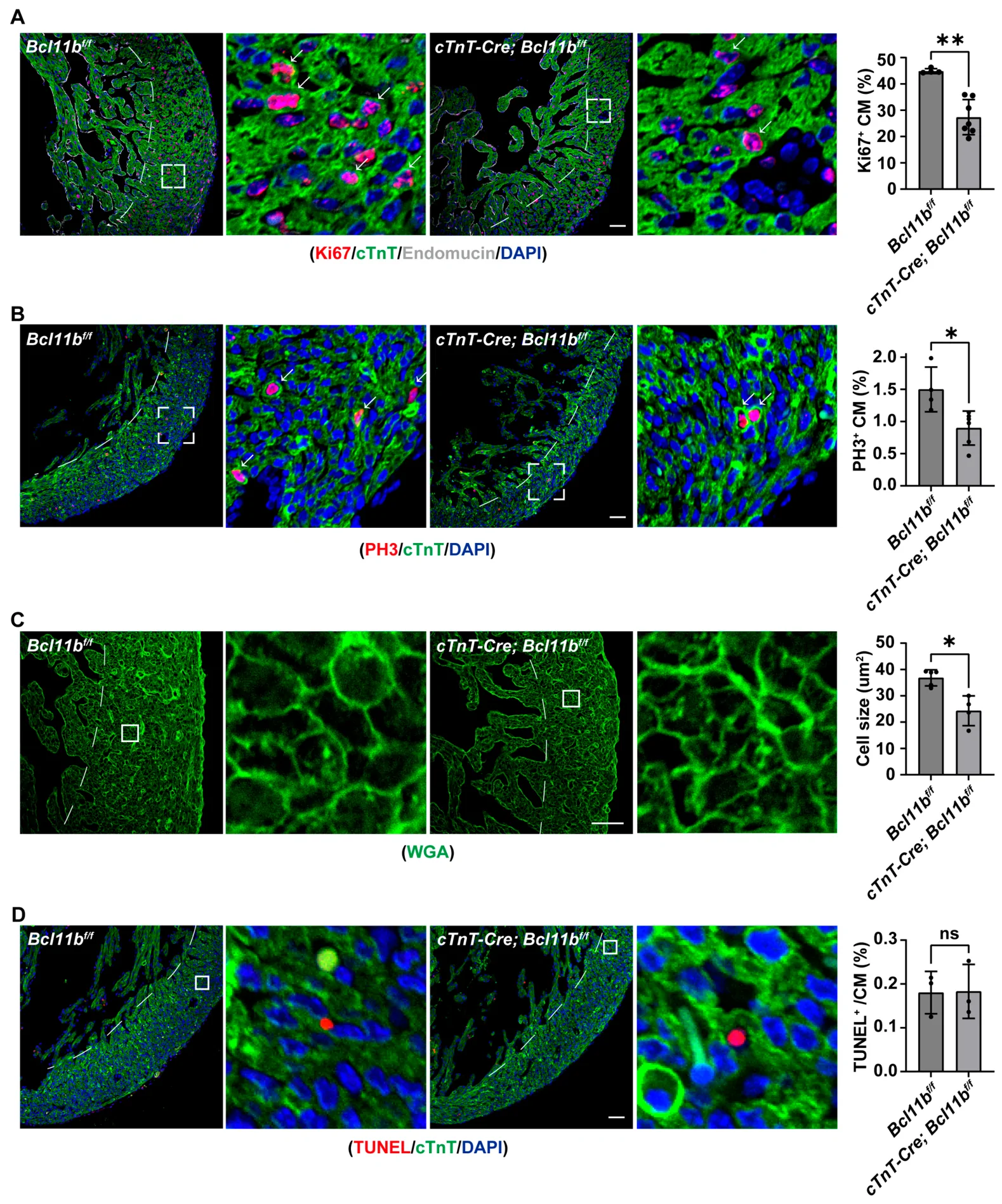
Cardiomyocyte-specific deletion of *Bcl11b* reduces proliferation and cell size without affecting apoptosis in LV compact layer. (**A**) Ki67^+^ cardiomoycytes in the LV cross sections from E16.5 *Bcl11b^f*/*f^* and *cTnT-Cre*; *Bcl11b^f*/*f^* mice. Ki67 (red), cTnT (green), Endomucin (gray), and DAPI (blue) staining are indicated. *n* = 4–7 hearts/group. (**B**) PH3^+^ cardiomoycytes in the LV cross sections from E16.5 *Bcl11b^f*/*f^* and *cTnT-Cre*; *Bcl11b^f*/*f^* mice. PH3 (red) staining is indicated. *n* = 4–6 hearts/group. (**C**) FITC-labeled (Green) wheat germ agglutinin (WGA) immunostaining for cardiomyocytes in the LV cross sections from E16.5 *Bcl11b^f*/*f^* and *cTnT-Cre*; *Bcl11b^f*/*f^* mice. *n* = 4 hearts/group. (**D**) TUNEL^+^ cardiomyocytes in the LV cross sections from E16.5 *Bcl11b^f*/*f^* and *cTnT-Cre*; *Bcl11b^f*/*f^* mice. TUNEL (red) staining is indicated. *n* = 3 hearts/group. Scale bar = 50 μm. Means ± SD are plotted; statistics were performed using Mann–Whitney U test (**A**,**C**) or Welch’s unpaired *t* test (**B**,**D**). * *p* < 0.05, ** *p* < 0.01; ns, non-significant.

Precise spatiotemporal regulation of CM proliferation and hypertrophic growth is essential for normal cardiac development [7,21] and disruption of these processes contributes to cardiac abnormalities, including LVNC [9,22]. We therefore investigated the effects of *Bcl111b* deletion on these developmental processes. As shown in Figure 2A,B, the percentages of Ki67-positive and phospho-histone H3 (PH3)-positive CMs were significantly reduced in CM-KO hearts. Consistently, WGA staining revealed a significant reduction in CM sizes in CM-KO hearts (Figure 2C). In contrast, no significant difference in CM apoptosis was observed between CM-KO and control hearts (Figure 2D). Collectively, these findings indicate that CM-specific deletion of *Bcl11b* impairs CM growth rather than survival, thereby contributing to defective ventricular compaction.

### 2.3. Bcl11b Suppresses Pou3f2 Expression in the CMs

**Figure 3:**
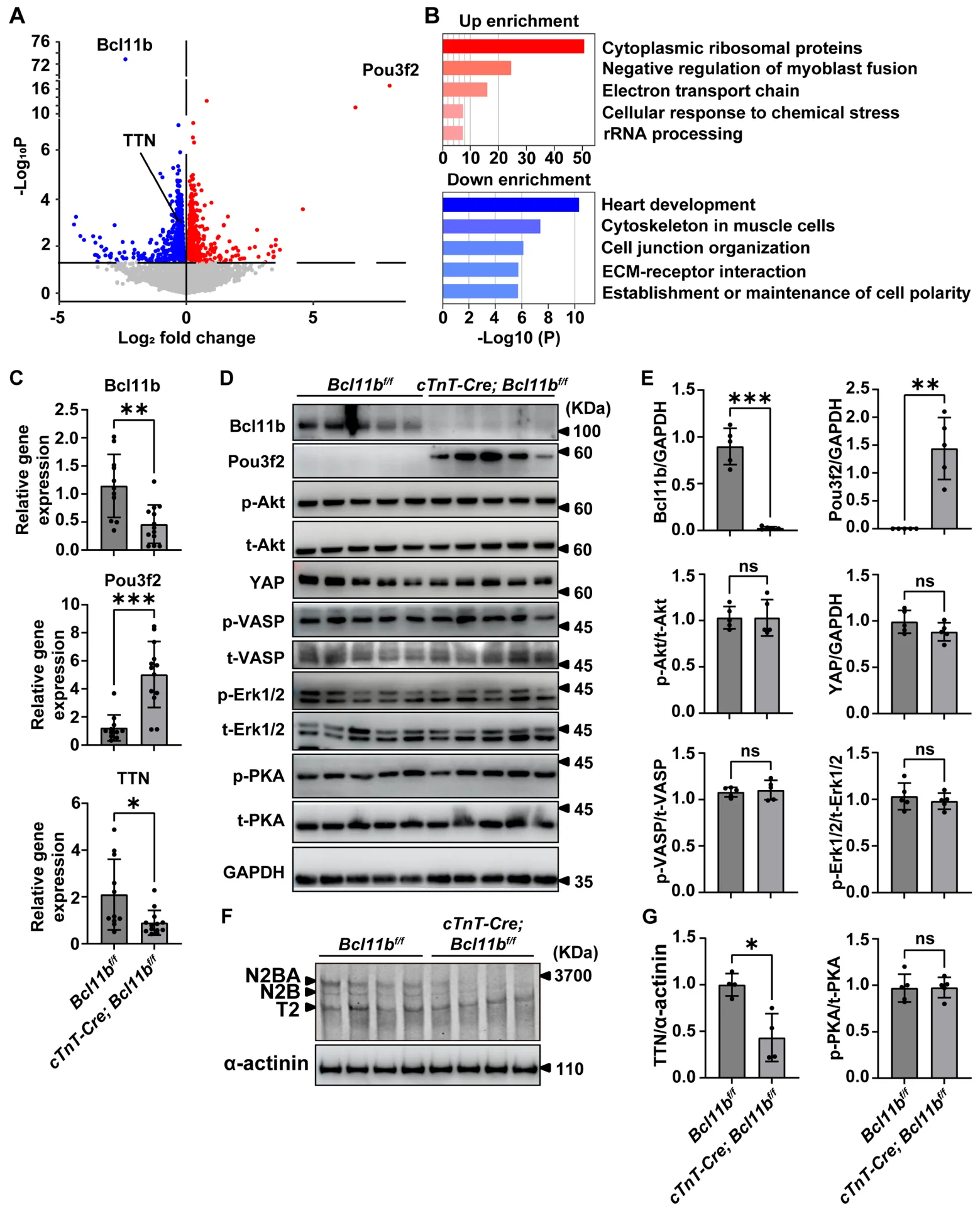
*Bcl11b* deficiency induces Pou3f2 upregulation while suppressing TTN expression in cardiomyocytes. (**A**) Volcano plot illustrating differentially up-(red) and down-(blue) regulated genes in heart ventricles of E16.5 *cTnT-Cre*; *Bcl11b^f*/*f^* mice compared with *Bcl11b^f*/*f^* controls. The horizontal dashed line indicates the statistical significance threshold corresponding to *p* = 0.05. *n* = 5 hearts/group. (**B**) GO enrichment analysis of differentially expressed genes. (**C**) qRT–PCR validation of gene expression changes identified by Bulk RNA-seq in the ventricles from E16.5 *Bcl11b^f*/*f^* and *cTnT-Cre*; *Bcl11b^f*/*f^* mice. *n* = 11–13 hearts/group. (**D**–**G**) Changes in Bcl11b and downstream proteins in the ventricles from E16.5 *Bcl11b^f*/*f^* and *cTnT-Cre*; *Bcl11b^f*/*f^* mice. *n* = 4–5 hearts/group. Means ± SD are plotted; statistics were performed using Mann–Whitney U test (**C**) or Welch’s unpaired *t* test (**E**,**G**). * *p* < 0.05, ** *p* < 0.01, *** *p* < 0.001; ns, non-significant.

Bcl11b functions as a transcription factor that regulates diverse cellular processes through transcriptional activation or repression during development and disease [23,24]. To elucidate the molecular mechanisms by which Bcl11b contributes to LVNC, we performed bulk RNA sequencing of left ventricular tissues from CM-KO and *Bcl11b^f*/*f^* littermate control mice at E16.5. Differential expression analysis identified 1128 upregulated genes and 1260 downregulated genes in CM-KO ventricles compared with controls (Figure 3A). Gene Ontology (GO) enrichment analysis revealed that upregulated genes were primarily associated with ribosome biogenesis, rRNA processing, and electron transport chain pathways, whereas the downregulated genes were significantly enriched in heart development, muscle cytoskeleton organization, cell junction organization, ECM–receptor interaction, and cell polarity, indicating that *Bcl11b* deficiency predominantly disrupts CM maturation and ventricular compaction-related developmental programs (Figure 3B).

Among the most significantly dysregulated genes, *Pou3f2* emerged as one of the top upregulated candidates following *Bcl11b* ablation. In parallel, *TTN,* a key sarcomeric gene implicated in LVNC pathogenesis in both humans and mice [25], was markedly downregulated. These transcriptional changes were further validated by qRT–PCR (Figure 3C). Notably, several signaling pathways implicated in CM development, including Akt, Erk1/2, and YAP, as well as previously reported downstream targets of Bcl11b, such as PKA and PKG [26,27,28], showed no significant alterations following CM-specific deletion of *Bcl11b* (Figure 3D,E and Figure S4). In contrast, Pou3f2 protein expression was markedly increased (Figure 3D,E), whereas TTN protein expression was substantially reduced (Figure 3F,G). Together, these findings suggest that *TTN* acts as a downstream effector of *Bcl11b-Pou3f2* regulatory network and contributes to the development of LVNC in this model.

### 2.4. Dysregulated Pou3f2 Impairs TTN Expression and Sarcomere Integrity

**Figure 4:**
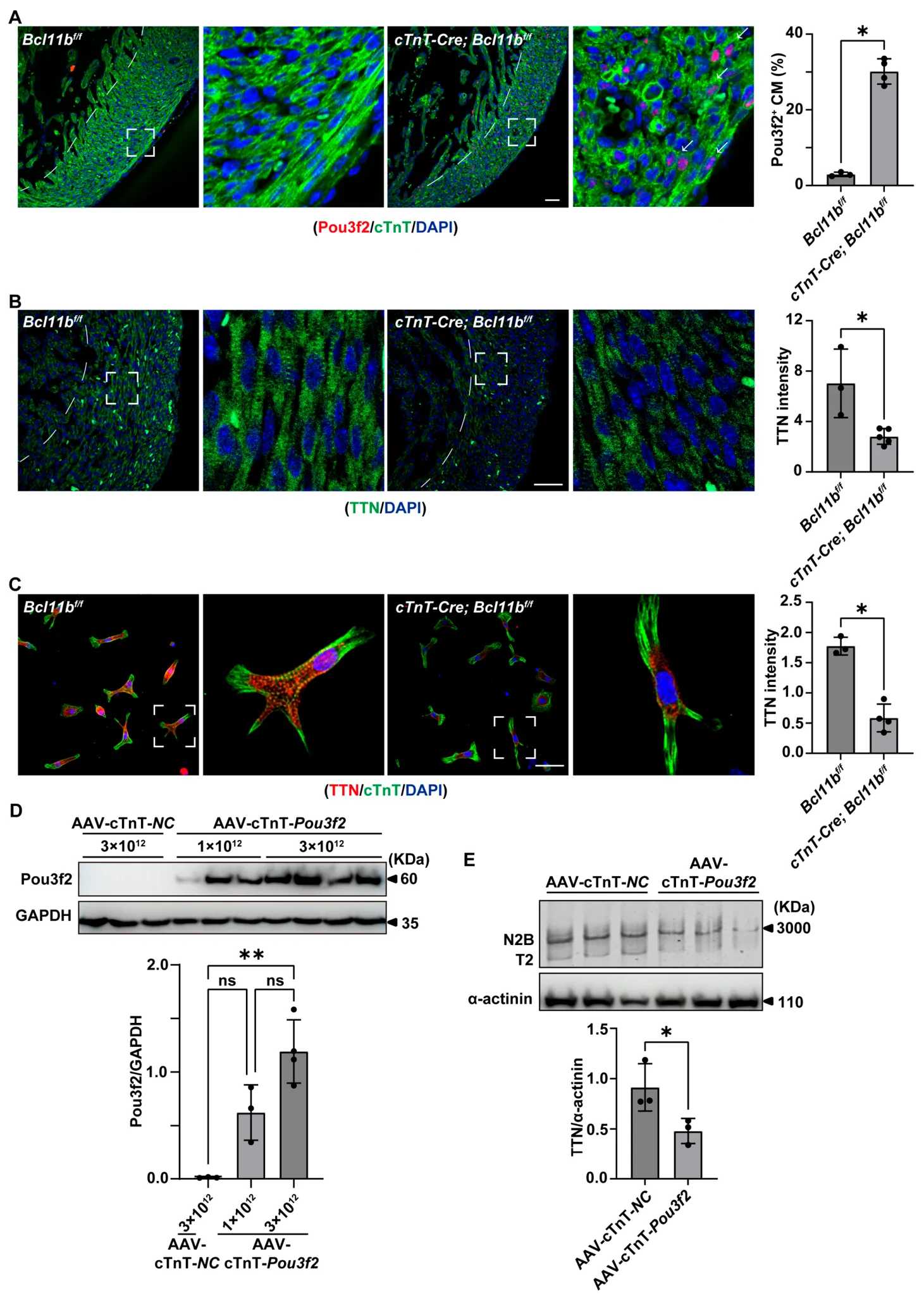
Upregulation of Pou3f2 inhibits normal TTN expression in CMs. (**A**) Pou3f2^+^ staining in the LV cross sections from E16.5 *Bcl11b^f*/*f^* and *cTnT-Cre*; *Bcl11b^f*/*f^* mice. Pou3f2 (red), cTnT (green), and DAPI (blue) staining are indicated. *n* = 3–4 hearts/group. (**B**) TTN staining (Green) in the LV cross sections from E16.5 *Bcl11b^f*/*f^* and *cTnT-Cre*; *Bcl11b^f*/*f^* mice. *n* = 3–5 hearts/group. (**C**) TTN staining (Red) in isolated CMs from E16.5 *Bcl11b^f*/*f^* and *cTnT-Cre*; *Bcl11b^f*/*f^* mice. *n* = 3–4. (**D**) Pou3f2 protein expression was overexpressed in the ventricles after intracardially injected AAV-cTnT-*Pou3f2*. *n* = 3–4 hearts/group. (**E**) TTN protein expression was suppressed in the ventricles after intracardially injected AAV-cTnT-*Pou3f2*. *n* = 3 hearts/group. Scale bar = 50 μm. Means ± SD are plotted; statistics were performed using Mann–Whitney U test (**A**–**C**), Welch’s unpaired *t* test or Welch’s ANOVA followed by multiple comparisons tests (**D**). * *p* < 0.05, ** *p* < 0.01.

Pou3f2 is a transcription factor implicated in the regulation of gene expression during development [29]. To investigate whether *Pou3f2* regulates *TTN* expression, we performed IF staining for Pou3f2 and TTN. In CM-KO hearts, nuclear Pou3f2 expression was markedly increased compared with *Bcl11b^f*/*f^* controls (Figure 4A). Consistently, the proportion of sarcomeric TTN-positive signal within the compact myocardium at E16.5 was significantly reduced in CM-KO hearts (Figure 4B). Moreover, TTN filament organization was markedly disrupted following *Bcl11b* knockdown in isolated CMs (Figure 4C), indicating impaired sarcomere integrity. Similarly, in cultured neonatal mice CMs (NMCMs), siRNA-mediated knockdown of *Bcl11b* significantly increased Pou3f2 expression compared with the negative control (*NC*) group (Figure S5A). IF analysis further confirmed enhanced nuclear Pou3f2 expression accompanied by loss of TTN integrity in the siRNA-*Bcl11b* group (Figure S5B,C).

To further investigate the functional role of Pou3f2, murine *Pou3f2* was overexpressed in WT mouse hearts using adeno-associated virus serotype 9 (AAV9). Quantification of AAV-derived GFP fluorescence showed that approximately 50% of CMs were successfully transduced (Figure S6). Western blot analysis demonstrated a dose-dependent increase in Pou3f2 protein expression following AAV9-cTnT-*Pou3f2* administration (Figure 4D). Consistent with these findings, TTN protein expression were significantly reduced 4 days after AAV9-cTnT-*Pou3f2* injection compared with the AAV9-cTnT-*NC* group (Figure 4E). Together, these findings support the hypothesis that *Pou3f2* negatively regulates *TTN* expression and thereby contributes to LVNC development.

### 2.5. Loss of Pou3f2 Rescues the LVNC Phenotype in Bcl11b-Deficient Mice

**Figure 5:**
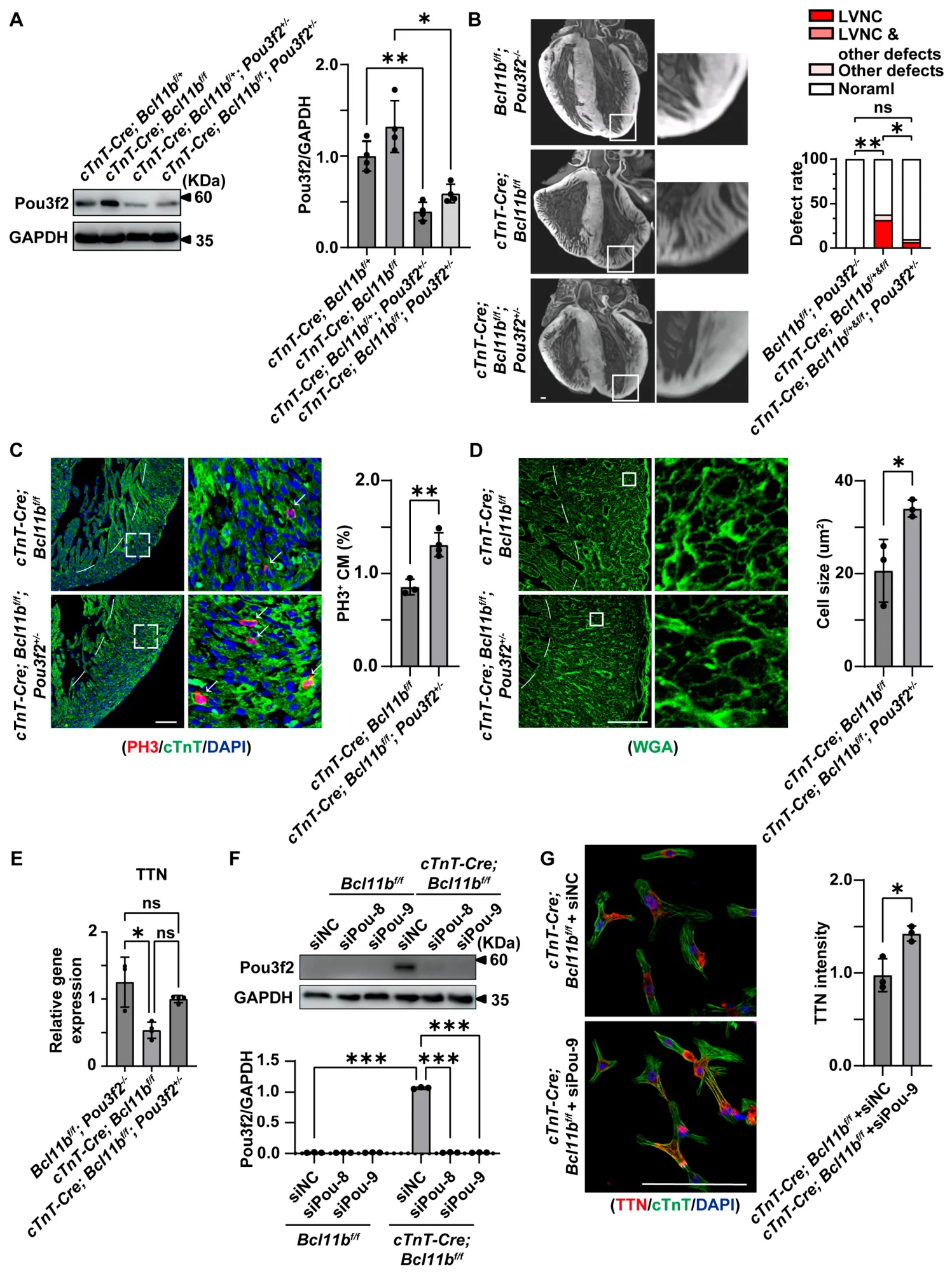
Inhibition of *Pou3f2* markedly rescues the LVNC phenotype resulting from *Bcl11b* deficiency. (**A**) Pou3f2 protein level in the ventricles from E16.5 CM-specific knockdown/knockout *Bcl11b* mice with *Pou3f2* knockdown or not. *n* = 4 hearts/group. (**B**) Morphology of E18.5 embryonic hearts from CM-specific knockout *Bcl11b* mice with *Pou3f2* knockdown or not. *n* = 16–31 hearts/group. (**C**) PH3^+^ staining in the LV cross sections from E16.5 *cTnT-Cre*; *Bcl11b^f*/*f^* and *cTnT-Cre*; *Bcl11b^f*/*f^*; *Pou3f2^+/−^* mice. PH3 (red), cTnT (green), and DAPI (blue) staining are indicated. *n* = 3–4 hearts/group. (**D**) FITC-labeled WGA immunostaining (Green) for cardiomyocytes in the LV cross sections from E16.5 *cTnT-Cre*; *Bcl11b^f*/*f^* and *cTnT-Cre*; *Bcl11b^f*/*f^*; *Pou3f2^+/−^* mice. *n* = 3 hearts/group. (**E**) qRT–PCR of TTN gene expression changes in the ventricles from E16.5 *Bcl11b^f*/*f^*, *cTnT-Cre*; *Bcl11b^f*/*f^* and *cTnT-Cre*; *Bcl11b^f*/*f^*; *Pou3f2^+/−^* mice. *n* = 3–4 hearts/group. (**F**) Changes in Pou3f2 protein level in isolated NMCMs from *Bcl11b^f*/*f^* and *cTnT-Cre*; *Bcl11b^f*/*f^* mice after treating with *siPou3f2* or *siNC*. *n* = 3. (**G**) TTN staining (Red) in isolated NMCMs from *Bcl11b^f*/*f^* and *cTnT-Cre*; *Bcl11b^f*/*f^* mice after treating with *siPou3f2* or *siNC*. *n* = 3. Scale bar = 100 μm. Means ± SD are plotted; statistics were performed using Welch’s unpaired *t* test (**C**,**D**,**G**), Kruskal–Wallis test (**E**), the chi-square test with Bonferroni correction for multiple comparisons (**B**), Welch’s ANOVA (**A**) or two-way ANOVA followed by multiple comparisons tests (**F**). * *p* < 0.05, ** *p* < 0.01, *** *p* < 0.001; ns, non-significant.

To further investigate the role of *Bcl11b-Pou3f2-TTN* regulatory network in LVNC development, we generated *Pou3f2* knockout (*Pou3f2^−/−^*) mice using CRISPER/Cas9-midiated genome editing and crossed them with *cTnT-Cre*; *Bcl11b^f/+^* mice. Western blot analysis demonstrated that Pou3f2 protein levels in E16.5 ventricular tissues were significantly reduced in *Pou3f2^+/−^*; CM-KO mice compared with *Pou3f2^+/+^*; CM-KO littermates (Figure 5A). Lightsheet imaging of E18.5 hearts revealed that *Pou3f2* haploinsufficiency markedly reduced the incidence of LVNC in *Bcl11b*-deficient hearts (Figure 5B). IF staining for PH3 (Figure 5C) and WGA (Figure 5D) at E16.5 further confirmed that *Pou3f2* haploinsufficiency restored the impaired CM growth caused by *Bcl11b* deletion, accompanied by a trend toward restoration of TTN expression (Figure 5E). Further echocardiographic analyses demonstrated that *Pou3f2* haploinsufficiency significantly improved cardiac systolic function in adult CM-specific Bcl11b knockout mice and restored ventricular wall thickness (Figure S7).

To further validate these findings in vitro, NMCMs isolated from CM-KO and *Bcl11b^f*/*f^* mice were transfected with *Pou3f2* siRNA. Knockdown of *Pou3f2* markedly attenuated the elevated Pou3f2 expression in *Bcl11b*-deficient NMCMs (Figure 5F) and restored TTN protein expression (Figure 5G). Consistently, co-transfection of *Pou3f2* siRNA with *Bcl11b* siRNA similarly restored TTN expression (Figure S8), which further supports a functional interaction between Bcl11b and Pou3f2 and their regulatory role on TTN expression during CM development.

## 3. Discussion

**Figure 6:**
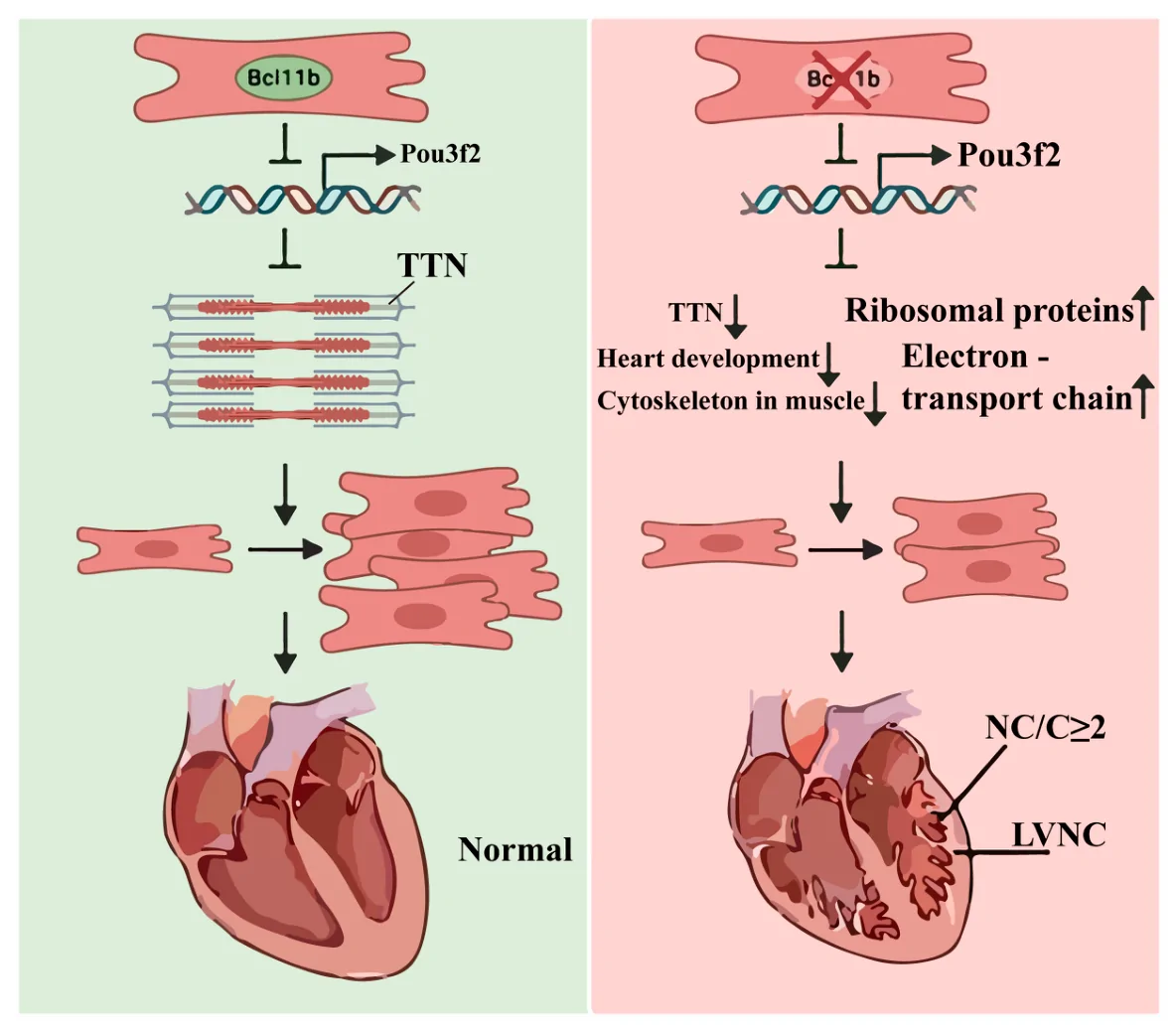
Proposed model of bcl11b deficiency in embryonic CMs leading to LVNC. (LVNC, left ventricular noncompaction; NC/C, noncompacted myocardium-to-compacted myocardium ratio).

A major conclusion of this study is that disruption of Bcl11b-Pou3f2-TTN regulatory network during heart development impairs CM growth and leads to LVNC (Figure 6). We demonstrate that cardiac Bcl11b maintains the formation of the compaction layer during embryogenesis. Mechanistically, Bcl11b functions, at least in part, by repressing expression of Pou3f2. Loss of Bcl11b in mice and NMCMs results in the upregulation and nuclear accumulation of Pou3f2, thereby suppressing TTN expression. Disruption of the Bcl11b–Pou3f2–TTN regulatory network consequently reduces CM number and growth during embryonic heart development, ultimately leading to impaired ventricular compaction.

LVNC is characterized by excessive trabeculation and inadequate myocardial compaction. Although genetic screening of LVNC patients has identified numerous gene mutations [30,31,32], the molecular and cellular mechanisms underlying the disorganization between ventricular trabeculation and compaction in LVNC remain poorly understood, partly owing to patient heterogeneity and the lack of suitable genetic models [33]. Beyond its well-established role in T-cell and neuronal development [23,34,35,36,37,38,39], Bcl11b functions as a transcriptional repressor by interacting with the RNA polymerase II-regulatory complex pTEFb at the MYH7 promoter in the hearts of mice with hypertrophic cardiomyopathy [40]. These findings suggest that Bcl11b may also contribute to the regulation of cardiac sarcomere organization and function. Here we demonstrated that specific knockout of Bcl11b in embryonic CMs causes an LVNC phenotype associated with impaired CM growth and disrupted TTN integrity in the compact myocardium.

Previous studies have reported hypertrabeculation in mice with endothelial cell-specific deletion of ADAMTS1 [41], Fkbp1a [10], Jarid2 [42,43], Ino80 [44], ptbp1 [45] and Nrg1 [6,46,47], which is generally accompanied by embryonic or neonatal lethality, with the exception of *Nfatc1-Cre*;*Ptbp1^fl*/*fl^* and *Tie2-Cre*;*Ptbp1^fl*/*f^* mice. In contrast, CM-specific deletion of Mib1 resulted in dilated hearts with a thin compact myocardium and enlarged noncompacted trabeculae, and notably, some mutant mice survived into adulthood [9,48]. Together, these findings suggest that reciprocal interactions between endothelial cells and CMs coordinate ventricular trabeculation and compaction, although the underlying mechanisms remain incompletely understood. Our findings further emphasize that cell type-specific gene expression and its temporal dynamics are critical determinants of LVNC pathogenesis. Several mechanisms including NOTCH, NRG1/ErbB and BMP have been proposed to control ventricular trabecular formation/maturation and disruption of these signaling pathways can lead to thin myocardium and/or LVNC. Trabecular growth and compaction requires controlled myocyte proliferation. Hence, Notch1 mutant decreased BMP10 [47] and loss of BMP10 results in dramatic reduction in proliferative activity in CMs, thin myocardium and defective trabecular growth [49]. Myocardial loss of the Notch ligand Jag1 caused reductions in compact myocardium proliferation and a thin compact myocardium [50]. On the other hand, hyperproliferation of trabecular myocardium as a result of either hyperactivation of endocardial Notch1 due to Fkbp1a mutation [10] or suppressed endocardial Notch1 activation Notch1 signaling following Mib1 deletion also leads to LVNC [9]. By extension, it is thought that defects in myocardial proliferation during embryonic heart development may contribute to the development of LVNC in human patients. However, pathogenic variants affecting these pathways are rarely identified in human LVNC patients. In our model, Bcl11b deletion results in reduced proliferation of CMs, accompanied by downregulation of genes involved in heart development and cytoskeleton in muscles, and upregulation of genes involved in protein translation and mitochondrial electron transport chain. The exact mechanism by which defective Bcl11b-Pou3f2 inhibit cell proliferation is unclear and does not seem to be related to p-AKT, p-ERK and Yap. The transcriptome suggests that Bcl11b might repress myofibril gene expression and muscle growth, which trigger an adaptive response to upregulate protein translation and respiration. Importantly, the survival of these mice into adulthood provides a valuable experimental platform for investigating disease progression and evaluating potential therapeutic strategies for LVNC.

Pou3f2 is highly expressed in neocortical progenitor cells during early embryonic brain development and plays essential roles in neural differentiation and cell fate determination [51,52,53,54]. Although the relationship between Bcl11b and Pou3f2 has not been fully elucidated, several lines of evidence suggest an antagonistic regulatory relationship between these two transcription factors. First, Bcl11b^+^ and Pou3f2^+^ cells display complementary expression domains in the developing brain, both in neurons differentiated from human hESCs [55] and in the mouse neocortex [56]. Second, Pou3f2 expression is reduced in Bcl11b^+^ cell population isolated from the frozen mouse neocortex by FIN-seq [57]. Third, in the somatosensory cortex of Pou3f3/2-cKO mice, the upper-layer markers RORβ and CUX1 are absent, accompanied by an increased number of cells expressing the deep-layer markers TLE4 and Bcl11b [58]. Our findings extend these observations by demonstrating that, in the heart, Bcl11b represses Pou3f2 expression to preserve TTN integrity and maintain normal CM growth. Pou3f2 overexpression was reported to repress proliferation in primary human NSCs [29] and in stem cell models of ASD [59,60], whereas Pou3f2 knockdown significantly increased the ratio of EdU-positive NPCs [61]. Consistent with these findings, both siRNA-mediated silencing and genetic ablation of *Pou3f2* mitigated the inhibitory effects of CM-specific Bcl11b deficiency on CM growth, supporting the conclusion that Pou3f2 functions as a negative downstream effector of Bcl11b in regulating CM growth. Although our study demonstrates that disruption of the Bcl11b–Pou3f2 regulatory axis leads to an LVNC-like phenotype in mice, we acknowledge that the underlying mechanisms of human LVNC may differ [62,63,64]. Human LVNC is more frequently associated with mutations in sarcomeric genes such as TTN, MYH7, and MYBPC3, suggesting a primary defect in myocardial contractility and mechanotransduction [64,65]. However, the Bcl11b–Pou3f2 pathway identified in this study is functionally connected to sarcomeric genes, such as TTN, and many other related proteins that may be potential downstream targets of this pathway. These findings raise the possibility that alterations in upstream transcriptional regulatory networks controlling sarcomeric gene expression may also contribute to the pathogenesis of human LVNC, in addition to pathogenic variants within sarcomeric genes themselves.

Numerous TTN mutations have been identified in patients with LVNC [16,65,66]. In addition, the dynamic changes in TTN isoforms during cardiac development and disease progression have been well characterized [25,67,68,69,70]. However, the functional significance and regulatory mechanisms of TTN expression changes during heart development remain incompletely understood. Furthermore, primary cultures of rat CMs undergo a transition from an undifferentiated mononuclear stage to a fully differentiated and contractile state, accompanied by the induction of TTN expression. However, when these cells are seeded at low density, TTN expression is completely downregulated [71], suggesting that TTN expression is closely associated with the balance between CM proliferation and maturation. In addition, increased TTN expression has been shown to reverse skeletal muscle atrophy in mice [72,73]. In our study, we identify TTN as a downstream target repressed by Pou3f2, providing a mechanistic link between Bcl11b deficiency and LVNC. Importantly, genetic ablation of Pou3f2 largely rescued the defects observed in Bcl11b-deficient mice, supporting the functional importance of this regulatory pathway in vivo. Collectively, these findings suggest that TTN acts as a critical downstream effector of the Bcl11b–Pou3f2 regulatory axis and highlight its essential role in coordinating CM growth and maturation during heart development. While the concomitant reduction in TTN transcripts is consistent with transcriptional regulation, we cannot exclude additional contributions from altered TTN isoform expression, changes in protein stability, or secondary impairment of sarcomere assembly. Further studies, including characterization of the relevant cis-regulatory elements, trans-acting factors, and transcription factor occupancy at the TTN locus, will be required to determine whether TTN is a direct transcriptional target of Pou3f2.

Therefore, although our data support TTN as an important downstream effector of the Bcl11b–Pou3f2 pathway, we cannot exclude the possibility that additional downstream targets also contribute to impaired ventricular compaction and CM maturation. Future studies involving TTN restoration will be required to determine whether re-establishing TTN expression alone is sufficient to rescue the phenotype. In addition, assessing Bcl11b and Pou3f2 expression in TTNtv patient-derived or engineered hiPSC-CMs may help determine whether dysregulation of this pathway also contributes in human LVNC. Furthermore, due to the ethical requirements governing animal studies and the limited availability of embryonic mouse samples, some experiments were performed with relatively small sample sizes, which represents a limitation of the present study. To address this limitation, statistical approaches were carefully selected based on data distribution characteristics and the assumptions of each statistical method.

Collectively, our findings identify the Bcl11b–Pou3f2–TTN regulatory network as a previously unrecognized mechanism governing CM growth and ventricular compaction during heart development, providing new insights into the molecular basis of LVNC.

## 4. Materials and Methods

### 4.1. Mice

All animal procedures were approved by the Institutional Animal Care and Use Committee of Westlake University and conducted in compliance with institutional guidelines (IACUC Protocol #25-102-SHJ and #26-008-CX). The *Bcl11b^flox*/*flox^* (*Bcl11b^f*/*f^*) mice (C57BL/6 background) were kindly provided by Shang Cai’s lab (Hangzhou, China), as previously reported [74,75]. The *Nkx2.5-Cre* and *cTnT-Cre* were purchased from Jackson Laboratory (Bar Harbor, ME, USA), and *nfatc1-Cre* mice were purchased from GemPharmatech (Nanjing, China).

*Pou3f2* knockout mice were generated using the Crispr-cas9 genome editing approach [76]. Briefly, single guide RNA (sgRNA) was designed using the IDT CRISPR tool (https://sg.idtdna.com/pages, accessed on 21 April 2022) and synthesized by GenScript. Equimolar amounts of sgRNA and Cas9 protein were mixed in RNase-free water to produce a microinjection solution with a final ribonucleoprotein (RNP) concentration of 2 µM. The RNP mixture was microinjected into C57BL/6J zygotes, which were subsequently transferred into pseudopregnant recipient females to produce founder mice. The sgRNA sequences and genotyping primers are listed in Supplementary Table S1. All mouse strains were maintained on a C57BL/6J genetic background for subsequent experiments. Adult mice were euthanized by cervical dislocation following deep anesthesia for tissue collection or by gradual-fill carbon dioxide inhalation when removed from the breeding colony, as appropriate. Embryonic and neonatal mice younger than postnatal day 7 (P7) were euthanized by decapitation.

### 4.2. Lightsheet Fluorescence Microscopy (LFM) and Morphological Analysis

Fetal hearts were harvested at embryonic stage day 18.5 (E18.5) and were imaged using the Zeiss Lightsheet Z. 7 microscope (Carl Zeiss Microscopy GmbH, Jena, Germany) as previously described [77]. In brief, hearts were fixed overnight in a mixed solution of 10% neutral buffered formalin and 2.5% glutaraldehyde after being dissected with phosphate-buffered saline (PBS), then rinsed twice in PBS, and dehydrated in 50%, 75%, and 100% ethanol in turn. Samples were transferred into a tube containing BABB solution for tissue clearing and mounted into the instrument chamber filled with 85% glycerol. Whole hearts were scanned and Imaris 10.2.0 software was used to carry out 3D reconstruction of the image stacks for morphological analysis. Heart morphology was assessed in 3D mode independently by two trained personnel.

### 4.3. Compact Layer and Non-Compact Layer Measurement

Lightsheet 3D images were analyzed using Imaris (version 10.2.0). The long-axis four-chamber view was used to measure the thickness of the compact myocardial layer and the non-compact myocardial layer. Papillary muscles, which were clearly identifiable and served as anatomical landmarks for the measurement plane, were excluded from the measurements. The compact myocardial layer was defined as the myocardial layer extending from the epicardial surface to the troughs of the trabeculae, showing homogeneous signal intensity. The non-compact myocardial layer was defined as a sponge-like myocardial layer with discontinuous, interspaced signal intensity. For each heart, thickness measurements were taken at five positions and averaged. The same anatomical locations were used consistently across all hearts.

### 4.4. Mice Echocardiography

P21 mouse transthoracic echocardiography was performed using a Vevo 3100 imaging system (VisualSonics Inc., Toronto, ON, Canada) equipped with an MS400 transducer. Mice were anesthetized with isoflurane (1% for maintenance), and body temperature was maintained at 37 °C. Chest hair was removed with depilatory cream, and ultrasound gel was applied to the thorax. Images were acquired when the heart rate stabilized between 450 and 510 beats per minute.

Left ventricle ejection fraction (LVEF) and left ventricle fractional shortening (LVFS) were measured in M-mode. Transmitral inflow velocities (E and A) were obtained by pulsed-wave Doppler, and myocardial velocities (E’ and A’) were measured using tissue Doppler imaging. All parameters represent the average of three consecutive cardiac cycles.

### 4.5. Immunofluorescence Staining and TUNEL Assay

Hearts were fixed in 4% paraformaldehyde (PFA) in PBS at 4 °C with gentle agitation on a rocker for 1–3 days, depending on tissue size. Samples were then dehydrated and embedded in paraffin (or OCT) following to standard laboratory procedures. Serial sections (7 µm) were prepared for immunofluorescence staining and TUNEL assays.

For immunofluorescence staining, sections were incubated in preheated sodium citrate buffer (pH 6.0) at 98 °C for 25 min for antigen retrieval. After blocking with 10% goat serum for 30 min at room temperature, sections were incubated with primary antibodies at 4 °C overnight, followed by incubation with appropriate Alexa Fluor-conjugated secondary antibodies for 1 h at room temperature. Sections were then counterstained with DAPI and mounted using VECTASHIELD antifade mounting medium (Vector Laboratories, Burlingame, CA, USA). Tyramide signal amplification (TSA) was used to enhance weak immunofluorescence signals (like Bcl11b and Pou3f2). After primary antibody incubation, sections were washed three times with 1 × TBST and incubated with HRP-conjugated secondary antibodies for 1 h at room temperature. Sections were then treated with amplification solution containing hydrogen peroxide and tyramide-biotin (1:200) for 8–10 min, followed by three washes. Finally, fluorophore-conjugated streptavidin and DAPI were applied for 1 h at room temperature before imaging.

After antigen retrieval, sections were washed with PBS and incubated with WGA for 10 min at room temperature, followed by PBS washes, DAPI staining, and mounting for imaging.

TUNEL staining was performed using the In Situ Cell Death Detection Kit (Roche Diagnostics GmbH, Mannheim, Germany), Fluorescein, according to the manufacturer’s instructions.

All Images were acquired using an Olympus FV3000 confocal microscope (Olympus Corporation, Tokyo, Japan). Three randomly selected microscopic fields in the left ventricle were analyzed for each biological sample, and the average value was used for statistical analysis.

### 4.6. RNA Isolation and Quantitative Real-Time PCR Assay

Total RNA was extracted from heart ventricle tissues using TRIzol Reagent (Invitrogen, Carlsbad, CA, USA). For each sample, 500 ng of RNA was reverse-transcribed into cDNA. Quantitative real-time PCR was performed using SYBR Green Mix (ABclonal, Wuhan, China) on a qTOWER real-time PCR system. Primer sequences are listed in Supplementary Table S1.

### 4.7. Western Blot Assay

Heart ventricle tissues were freshly excised and were snap frozen in liquid nitrogen, then homogenized with homogenizer in tissue lysis buffer (20 mM Tris–HCl, pH 7.4; 150 mM NaCl; 2.5 mM EDTA; 1% Triton X-100; 10% glycerol; 0.1% SDS; 0.5% sodium deoxycholate) supplemented with protease inhibitor and phosphatase inhibitor (1:200) on ice. For cultured primary cardiomyocytes (CMs), the cells were washed by ice-cold PBS and lysed by sonication in RIPA buffer (50 mM Tris–HCl, pH 7.4; 150 mM NaCl; 1 mM EDTA; 1% Triton X-100; 0.1% SDS; 1% sodium deoxycholate) supplemented with protease inhibitor and phosphatase inhibitor on ice. Tissue and cell lysates were centrifuged at 13,000 rpm for 30 min at 4 °C to remove insoluble material. Electrophoresed and blotted were performed by standard protocols.

### 4.8. Neonatal Mice CMs (NMCMs) Isolation and Manipulation

NMCMs were isolated from neonatal hearts as previously described [78] with minor modifications. Briefly, neonatal mice were decapitated, and hearts were rapidly excised and transferred into ice-cold calcium- and magnesium-free HBSS under sterile conditions. After removal of lung tissue, major vessels and blood clots, the hearts were mechanically dissociated into small fragments. Tissues were pre-digested in 0.25% trypsin diluted in HBSS at 4 °C for ~10 h. The digestion was terminated by adding complete medium (DMEM/F-12 supplemented with 10% FBS, 100 U/mL penicillin, and 100 μg/mL streptomycin), followed by incubation at 37 °C for 10 min. After removal of the supernatant, tissues were further digested with collagenase II (1%) in DMEM/F-12 at 37 °C with gentle agitation until complete dissociation. The cell suspension was filtered through a 70 μm cell strainer and centrifuged at 300× *g* for 5 min at room temperature. The pellet was resuspended in complete medium and plated onto 10 cm culture dishes for pre-plating at 37 °C for 1.5–2 h to allow preferential attachment of non-myocytes (mainly fibroblasts). The supernatant enriched in CMs was collected and either processed for protein analysis or used for subsequent culture.

For CM culture, plates were pre-coated with collagen. Cells were seeded at a density of approximately 2 × 10^5^ cells/mL and maintained in complete medium at 37 °C. After 24 h, the medium was replaced with fresh DMEM/F-12. For siRNA transfection, NMCMs were transfected with specific siRNAs using RNAiMAX (Carlsbard, CA, USA) according to the manufacturer’s instructions. Cells were incubated for 1–3 days prior to downstream analyses. All cell experiments were independently repeated at least three times.

For immunofluorescence, cells were fixed with 4% paraformaldehyde at room temperature. All images were acquired using an Olympus FV3000 confocal microscope. In total, 3–5 randomly selected pictures were taken for each group, and the average value was used for statistical analysis.

For protein analysis, cells were lysed in RIPA buffer on ice for subsequent Western blot experiments.

### 4.9. Bulk RNA Sequencing and Data Analysis

Embryonic hearts at day 16.5 were harvested in cold PBS, and ventricular tissues were dissected and immediately placed in cold TRIzol reagent (Invitrogen). Total RNA was isolated following the manufacturer’s instructions. mRNA-seq libraries were prepared using Fast RNA-seq Library Prep Kit V2 (Cat. No. RK20306;ABclonal). Sequencing was performed on the Illumina Novaseq 6000 platform (Illumina, San Diego, CA, USA) using a paired-end 150bp (PE150) strategy.

### 4.10. Adeno-Associated Viruses 9-pou3f2 Overexpression Assay

AAV9-*pou3f2* overexpression and control viruses were purchased from PackGene (Guangzhou, China). The AAV9-*pou3f2* overexpression vector expressed Pou3f2 and EGFP under the control of the cardiomyocyte-specific cTnT promoter, whereas the control vector expressed EGFP alone driven by the same promoter. C57BL/6 mice were intracardially injected at postnatal day 3 (P3) with either the overexpression or control virus at a dose of 1 × 10^12^–3 × 10^12^ viral genomes (vg)/per mouse as previously described [79]. Cardiac-specific transduction efficiency was assessed at day 4 after injection by fluorescence imaging and Western blot analysis.

### 4.11. Statistical Analysis

Results are presented as mean ± SD. Each dot represents an individual biological replicate, including individual animal samples or independent experiments, as specified in each figure legend. Data distribution was assessed using the Shapiro–Wilk test, and variance homogeneity was considered when selecting parametric tests. For datasets that met the assumptions for parametric analyses, Welch’s *t*-test, one-way ANOVA, or two-way ANOVA were applied as appropriate. For datasets that did not satisfy these assumptions, non-parametric tests were used. Being statistically significant was considered when *p*-value < 0.05.

## Data Availability

Further information and requests for resources should be directed to and will be fulfilled by the lead contact, Hongjun Shi (shihongjun@westlake.edu.cn). Materials and resources listed in the key resources table are available upon request.

## Supplementary Materials

The following supporting information can be downloaded at: https://www.mdpi.com/article/10.3390/ijms27167199/s1.

## Abbreviations

The following abbreviations are used in this manuscript:

AAV9	Adeno-associated virus serotype 9
Bcl11b	B-cell leukemia/lymphoma 11b
CHD	Congenital heart disease
CMs	Cardiomyocytes
cTnT	Cardiac troponin T
DCM	Dilated cardiomyopathy
Erk1/2	Extracellular regulated kinase 1/2
GAPDH	Glyceraldehyde-3-phosphate dehydrogenase
HCM	Hypertrophic cardiomyopathy
HF	Heart failure
LVEF	Left ventricular ejection fraction
LVFS	Left ventricular fractional shortening
LVNC	Left ventricular noncompaction cardiomyopathy
NC	Negative control
NKX2.5	NK2 homeobox 5
NOTCH1	Notch receptor 1
PH3	Phospho-histone H3
Pou3f2	POU class 3 homeobox 2
TTN	Titin/connectin
WT	Wild type
