# Supplementary material for: Cardiomyocyte-Specific bcl11b Knockout Causes Left Ventricular Noncompaction by Dysregulating pou3f2 and Titin
Source: Int J Mol Sci. 2026 Aug 12;27(16):7199. doi: 10.3390/ijms27167199 (PMC13513006; doi:10.3390/ijms27167199)
Supplement: Supplementary file 1 [file ijms-27-07199-s001.zip › ijms-4401005-supplementary-pub.pdf]

Supplemental information

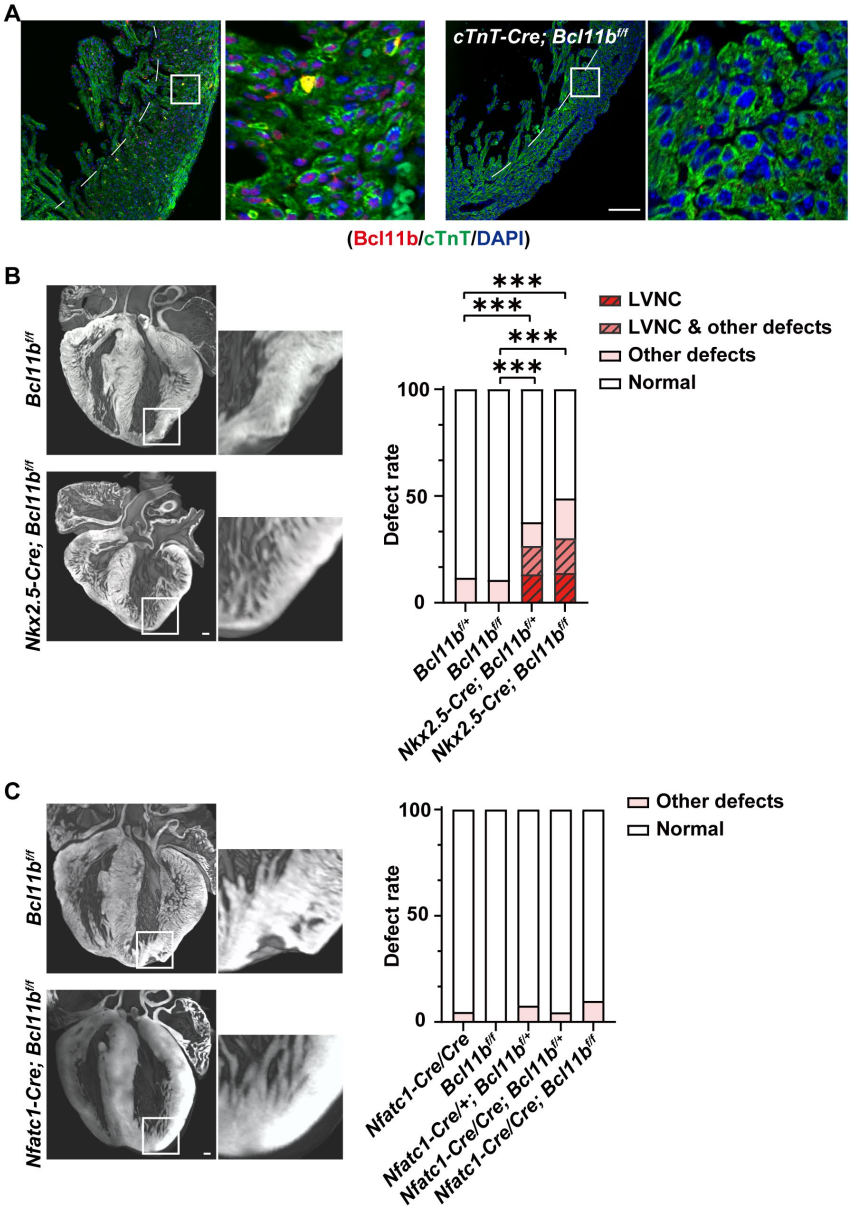

D

	Bcl11b$^{f/f}$	*Bcl11b$^{f/f}$*	*Nkx2.5-Cre; Bcl11b$^{f/+}$*	*Nkx2.5-Cre; Bcl11b$^{f/f}$*	Statistics
Survival (n)	45	46	27	25	X-squared=10.706, df=3, p-value=0.01342

	Bcl11b$^{f/f}$	*Bcl11b$^{f/f}$*	*cTnT-Cre; Bcl11b$^{f/+}$*	*cTnT-Cre; Bcl11b$^{f/f}$*	Statistics
Survival (n)	33	33	39	30	X-squared=1.5882, df=3, p-value=0.6621

Figure S1. Bcl11b expression levels after cardiomyocyte-specific knockout and heart defect incidence following *Bcl11b* deletion and, relative to Figure 1.

(A) Bcl11b staining in heart sections from *Bcl11b$^{f/f}$* and *cTnT-Cre; Bcl11b$^{f/f}$* mice.

(B) Morphology of E18.5 embryonic hearts from *Bcl11b*$^{f/f}$ and *Nkx2.5-Cre; Bcl11b*$^{f/f}$ mice. N=43-65 hearts/group.

(C) Morphology of E18.5 embryonic hearts from *Bcl11b*$^{f/f}$ and *Nftac1-Cre; Bcl11b*$^{f/f}$ mice. N=7-22 hearts/group.

(D) Survival analysis of mice within the first 3 days after birth.

Scale bar = 100 μm. Means±SD are plotted; statistical analysis was performed using the chi-square test with Bonferroni correction for multiple comparisons. ***$p < 0.001$.

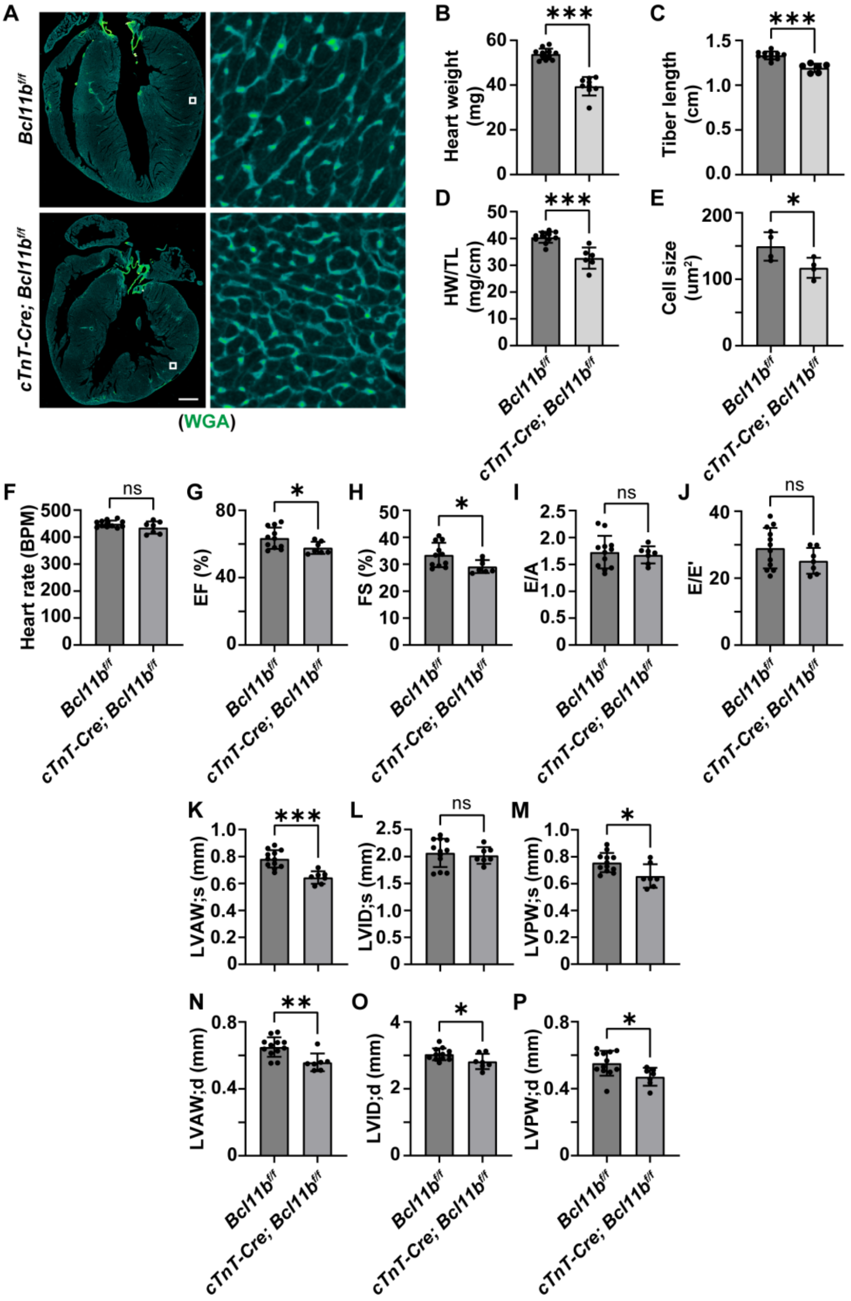

Figure S2. Postnatal development of cardiomyopathy in CM-specific *Bcl11b* knockout mice, relative to Figure 1.

(A) WGA staining of cardiac sections from postnatal day 21 (P21) *Bcl11b*$^{f/f}$ and *cTnT-Cre; Bcl11b*$^{f/f}$ mice (the right panel shows a magnified view of the boxed region).

(B-D) Quantification analysis of heart weight (HW), tibia length (TL), and the HW/TL ratio from P21 *Bcl11b*$^{f/f}$ and *cTnT-Cre; Bcl11b*$^{f/f}$ mice. N=9-11 hearts/group.

(E) Quantitative analysis of cardiomyocyte size in cardiac sections from P21 *Bcl11b*$^{f/f}$ and *cTnT-Cre; Bcl11b*$^{f/f}$ mice. N=4 hearts/group.

(F-P) Echocardiographic assessment of heart rate, LV ejection fraction (LVEF), LV fractional shortening (LVFS), E/A and E/E' ratios, as well as left ventricular anterior wall thickness (LVAW), internal diameter (LVID), and posterior wall thickness (LVPW) at systole (s) and diastole (d) in P21 *Bcl11b*$^{f/f}$ and *cTnT-Cre; Bcl11b*$^{f/f}$ mice. N=7-11 hearts/group.

Scale bar = 500 μm. Means±SD are plotted; statistics of were performed using Welch's unpaired t (E), two-tailed unpaired Student's t test (A-D, F-P). **p* < 0.05, ***p* < 0.01, ****p* < 0.001; ns, non-significant.

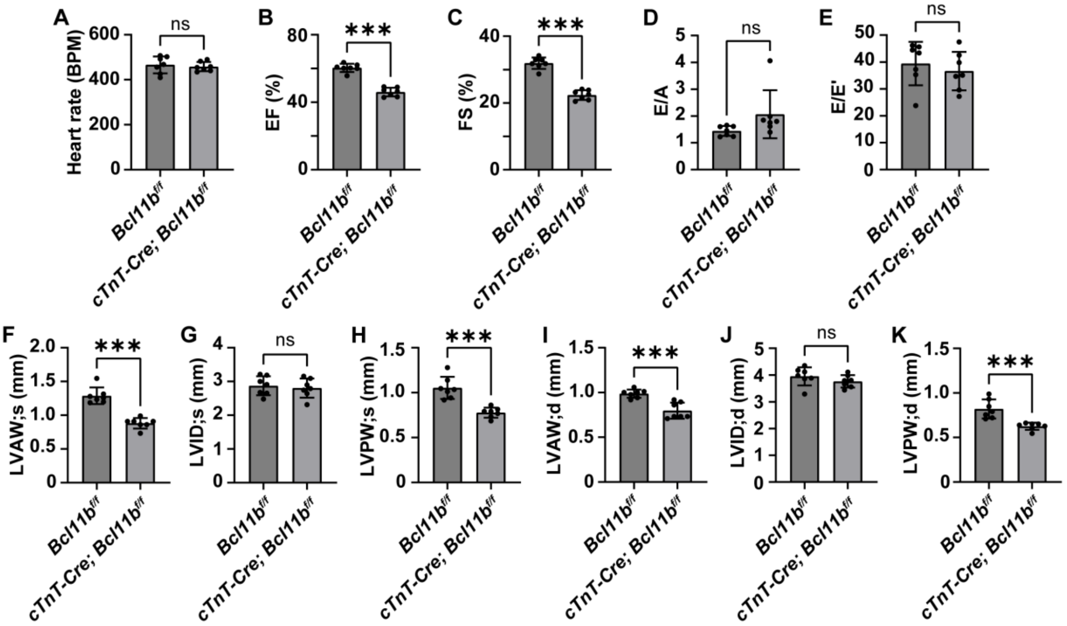

Figure S3. Functional characterization of adult CM-specific *Bcl11b* knockout mice, relative to Figure 1.

(A-K) Echocardiographic assessment of heart rate, LVEF, LVFS, E/A and E/E' ratios, as well as LVAW, LVID, and LVPW at systole (s) and diastole (d) in 8-10w *Bcl11b*$^{f/f}$ and *cTnT-Cre; Bcl11b*$^{f/f}$ mice. N=7 hearts/group.

Means±SD are plotted; statistics of were performed using two-tailed unpaired Student's t test. ****p* < 0.001; ns, non-significant.

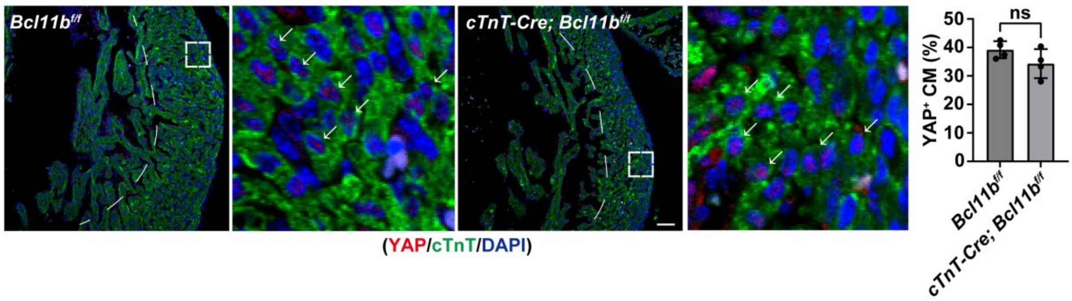

Figure S4. CM-specific *Bcl11b* knockout does not affect YAP expression in the heart, relative to Figure 3.

YAP staining in heart sections from E16.5 *Bcl11b*$^{f/f}$ and *cTnT-Cre; Bcl11b*$^{f/f}$ mice. N=4 hearts/group. Scale bar = 50 μm. Means±SD are plotted; statistics of were performed using Welch's unpaired t. ns, non-significant.

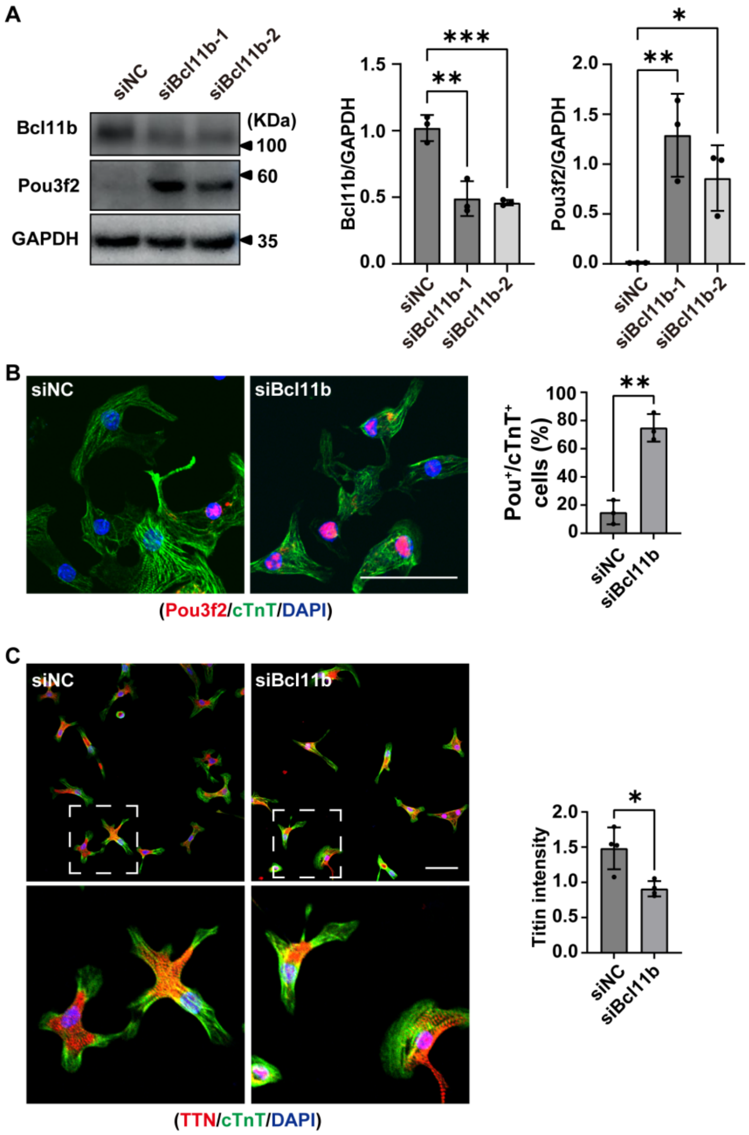

Figure S5. siRNA-mediated depletion of *Bcl11b* induces Pou3f2 upregulation, accompanied by further suppression of TTN expression in NMCMs, relative to Figure 4.

(A) Changes of Bcl11b and Pou3f2 protein level in isolated NMCMs from wild type (WT) mice after treating with *siBcl11b* or *siNC*. N=3.

(B) Pou3f2^{+} staining in isolated NMCMs from WT mice after treating with *siBcl11b* or *siNC*. N=3.

(C) TTN staining in isolated NMCMs from WT mice after treating with *siBcl11b* or *siNC*. N=4.

Scale bar = 50 μm. Means±SD are plotted; statistics of were performed using Welch's unpaired t (B, C), one-way ANOVA followed by multiple comparisons tests (A). *$p < 0.05$, **$p < 0.01$, ***$p < 0.001$.

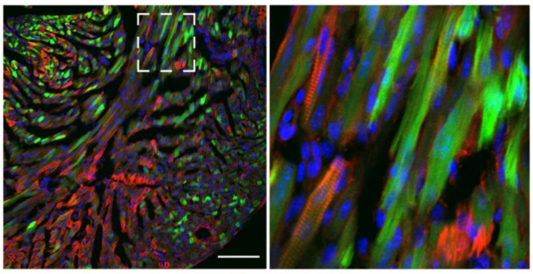

(cTnT/EGFP/DAPI)

Figure S6. Validation of the efficiency and specificity of AAV-mediated transduction in cardiomyocytes, relative to Figure 4.

Representative immunofluorescence images showing the co-localization of cTnT and the AAV9-encoded GFP.

Scale bar = 100 μm.

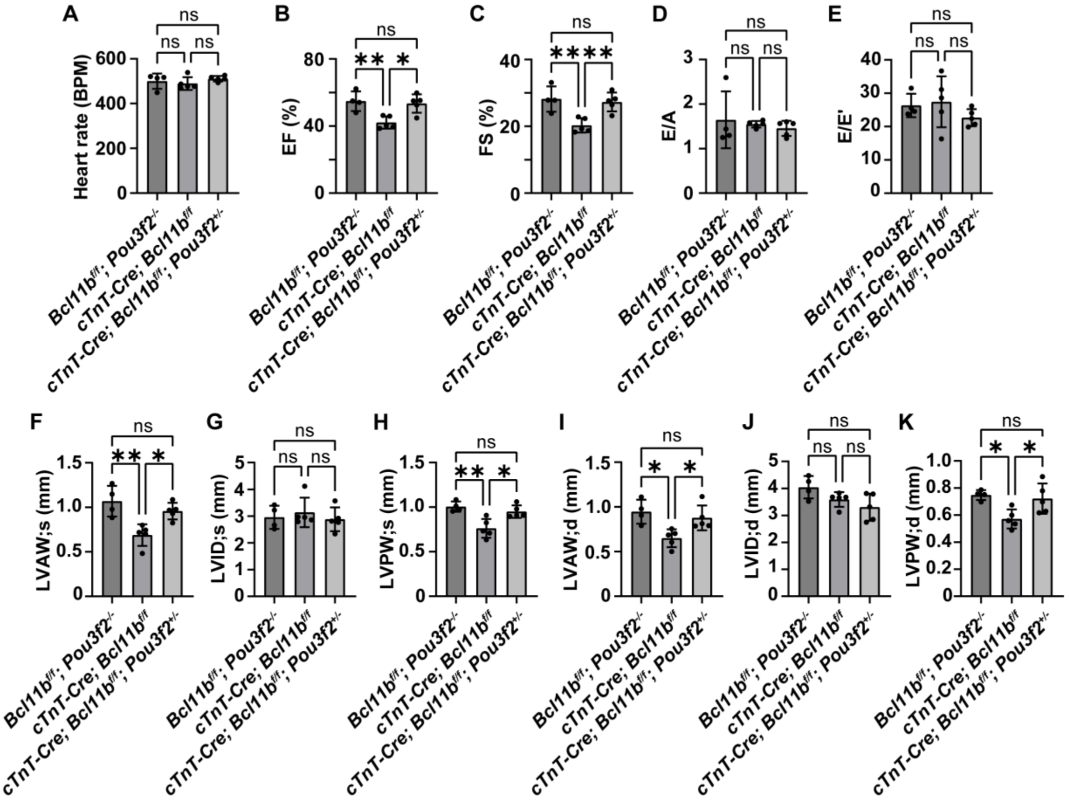

Figure S7. ***Pou3f2*** **haploinsufficiency restores cardiac function in adult CM-specific** ***Bcl11b*** **knockout mice, relative to Figure 5.**

(A-K) Echocardiographic assessment of heart rate, LVEF, LVFS, E/A and E/E' ratios, as well as LVAW, LVID, and LVPW at systole (s) and diastole (d) in 8-10w *Bcl11b*$^{f/f}$ and *cTnT-Cre; Bcl11b*$^{f/f}$ mice. N=4-5 hearts/group.

Means±SD are plotted; statistics of were performed using one-way ANOVA followed by multiple comparisons tests. *$p < 0.05$, **$p < 0.01$, ***$p < 0.001$; ns, non-significant.

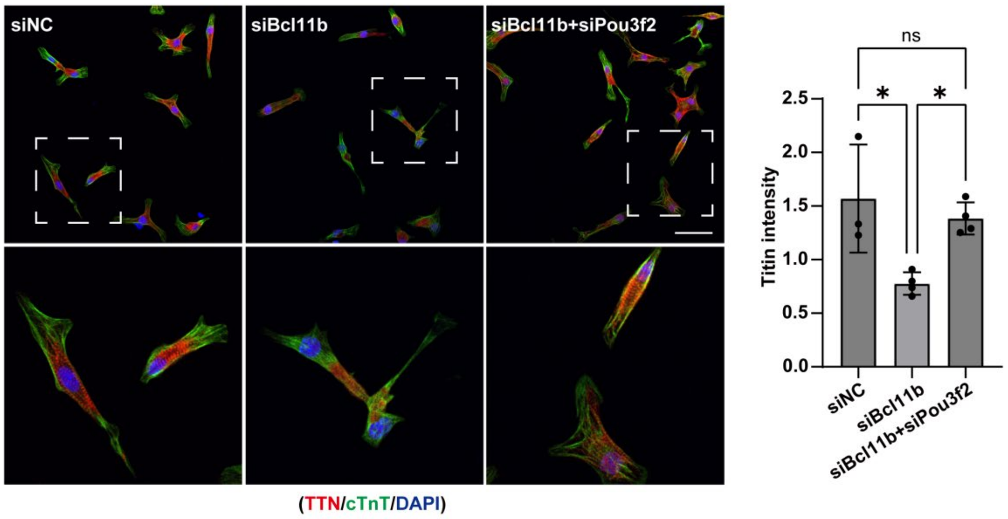

Figure S8. siRNA-mediated inhibition of *Pou3f2* rescues the downregulation of TTN expression caused by *Bcl11b* knockdown, relative to Figure 5.

TTN staining in isolated NMCMs from WT mice after treating with *siBcl11b+siPou3f2*, *siBcl11b* or *siNC*. N=3-4.

Scale bar = 50 μm. Means±SD are plotted; statistics of were performed using one-way ANOVA followed by multiple comparisons tests. *$p < 0.05$; ns, non-significant.

Table S1. Sequences of the oligonucleotides, related to STAR methods

**Primers for qRT-PCR**	
Bcl11b Forward	5’- AGGAGAGTATCTGAGCCAGTG-3’
Bcl11b Reverse	5’- GTTGTGCAAATGTAGCTGGAAG-3’
Pou3f2 Forward	5’- GTTGCCGTTTTGGGGGATTT-3’
Pou3f2 Reverse	5’- AATGGAGAGTGGCCAAGAGC-3’
TTN Forward	5’-AGAAGAGCCTAGCAGCCTGG-3’
TTN Reverse	5’-TGAGCCCCATCATGCAGACC-3’
Gapdh Forward	5’-CATCACTGCCACCCAGAAGACTG-3’
Gapdh Reverse	5’-ATGCCAGTGAGCTTCCCGTTCAG-3’
**Sequences of sgRNA used to generate *Pou3f2*$^{-/-}$ mice**	
GCTGTAGTGGTTAGACGCTG	
**Continued**	
**Sequences of siRNA used in vitro**	
NC-s	ACGUGACACGUUCGGAGAA/dT//dT/
NC-a	UUCUCCGAACGUGUCACGU/dT//dT/
mBcl11b-1s	CGCAGGCGAGCAAGCUCAA/dT//dT/
mBcl11b-1a	UUGAGCUUGCUCGCCUGCG/dT//dT/
mBcl11b-2s	GCGGCAAGGUCUUCAAGAA/dT//dT/
mBcl11b-2a	UUCUUGAAGACCUUGCCGC/dT//dT/
mPou3f2-8s	GCAGCGUCUAACCACUACA/dT//dT/

mPou3f2-8a	UGUAGUGGUUAGACGCUGC/dT//dT/
mPou3f2-9s	CCACCAGCAUAGACAAGAU/dT//dT/
mPou3f2-9a	AUCUUGUCUAUGCUGGUGG/dT//dT/
**Genotyping primers**	
Bcl11b-flox Forward	5'-AGTTTGCCGGAAGACTCTCTC-3'
Bcl11b-flox Reverse	5'-GGGTGCTCTGGTAATTTTCCTTA-3'
Cre Forward	5'-GTGGCAAAGTGGAGATTGTTG-3'
Cre Reverse	5'-CTCCTGGAAGATGGTGATGG-3'
Pou3f2-wt-Forward	5'-CAGGCTGTAGTGGTTAGACGCTG-3'
Pou3f2-mutant-Reverse	5'-CGAGAGTCATGGCGACCGCTA-3'
Pou3f2-common-Reverse	5'-GCAACAGAAGGCGTCGGAGC-3'

Table S2. KEY RESOURCES TABLE

REAGENT or RESOURCE	SOURCE	IDENTIFIER
Antibodies		
Anti-Pou3f2	CST	Cat# 12137
Anti-Erk1/2	CST	Cat# 4695; RRID: AB_ 390779
Anti-p-Erk1/2	CST	Cat# 4370; RRID: AB_ 2315112
Anti-Akt	CST	Cat# 4691; RRID: AB_ 915783
Anti-p-Akt	CST	Cat# 4060; RRID: AB_ 2315049
Anti-YAP	CST	Cat# 14074; RRID: AB_2650491
Anti-VASP	CST	Cat# 3132; RRID: AB_2213393
Anti-PKA	CST	Cat# 5842; RRID: AB_10706172
Anti-p-PKA	CST	Cat# 5661; RRID: AB_10707163
Anti-p-VASP	Affbiotech	Cat# AF3338; RRID: AB_2834753
**Continued**		
Anti-GAPDH	ABclonal	Cat# AC033
Anti-PH3	ABclonal	Cat# AP0840SP
Anti-tubulin	DSHB	Cat# AB528499
Anti-cTnT	DSHB	Cat# AB2240831
Anti-Bcl11b	Abcam	Cat# ab18465
Anti-endomucin	Santa cruz	Cat# sc-69495
Alexa Fluor 488 Donkey anti-mouse	Jackson	Cat# 715-545-150
Cy3 AffiniPure Donkey anti-Rabbit	Jackson	Cat# 711-167-003
Cy5 AffiniPure Donkey anti-Rat	Jackson	Cat# 711-607-003
Cy3 Streptavidin	Jackson	Cat# 016-160-084
Anti-Rabbit secondary antibody/HRP	CST	Cat# 7074
Anti-Mouse secondary antibody/HRP	CST	Cat# 7076

DAPI	Beyotime	Cat# C1002
WGA	Biotium	Cat# 29022
Chemicals, peptides, and recombinant proteins		
DMEM/F12	Gibco	Cat# C11330500BT
FBS	Solarbio	Cat# S9030
Trypsin-EDTA	Cellmax	Cat# CPT101.02
HBSS	Gibco	Cat# C14175500BT
Collagenase II	Worthington	Cat# LS004176
Collagen	Gibco	Cat# S006100
Isoflurane	RWD	Cat#
PFA	Sigma	Cat# P6148
Triton X-100	Macklin	Cat# I997471
REGENT or RESOURCE	SOURCE	IDENTIFIER
DMSO	Macklin	Cat# D806645
Citrate Antigen Retrieval Solution	Beyotime	Cat# P0083
Protease inhibitor cocktail	MCE	Cat# HY-K0010
Phosphatase Inhibitor Cocktail	MCE	Cat# HY-K0022
RNAiMAX	Invitrogen	Cat# 56532
Critical commercial assays		
TRIzol reagent	Invitrogen	Cat# 15596018CN
In situ Cell Death Detection Kit, POD	Roche	Cat# 11684795910
SYBR Green Mix	ABclonal	Cat# RM21217
Enhanced BCA Protein Assay Kit	Beyotime	Cat# P0010S
BeyoECL Moon	Beyotime	Cat# P0018FM
TIANgel Midi Purification Kit	TIANGEN	Cat# DP209-03
FastKing RT-PCR MasterMix	TIANGEN	Cat# KR123
**Continued**		
Experimental models: Organisms/strains		
Mouse: *Nkx2.5-Cre*	Jax	Cat# 03004
Mouse: *Nfatc1-Cre*	GemPharmatech	Cat# T006813
Mouse: *cTnT-Cre*	Jax	Cat# 024240
Mouse: *Pou3f2*$^{-/-}$	This paper	N/A
Recombinant DNA		
AAV9-cTnT-NC/Pou3f2	PackGene	N/A
Software and algorithms		
GraphPad Prism v10.1	GraphPad Softwave Inc	N/A
ImageJ	ImageJ	https://imagej.net
Imaris v10.2.0	Bitplane	N/A

Olympus FV3000	Olympus	N/A
Vevo Lab 5.7.1	FUJIFILM	N/A
